# *Stand More AT Work (SMArT Work)*: using the behaviour change wheel to develop an intervention to reduce sitting time in the workplace

**DOI:** 10.1186/s12889-018-5187-1

**Published:** 2018-03-06

**Authors:** Fehmidah Munir, Stuart J. H. Biddle, Melanie J. Davies, David Dunstan, David Esliger, Laura J. Gray, Ben R. Jackson, Sophie E. O’Connell, Tom Yates, Charlotte L. Edwardson

**Affiliations:** 1 School of Sport, Exercise and Health Sciences, Loughborough University, Leicestershire, UK; 2 Institute for Resilient Regions, University of Southern Queensland, Springfield Central, Australia; 3 Diabetes Research Centre, University of Leicester, Leicester, UK; 4 Leicester Diabetes Centre, University Hospitals of Leicester, Leicester, UK; 5 NIHR Leicester Biomedical Research Centre, Leicester, UK; 6 School of Public Health, The University of Queensland, Brisbane, QLD Australia; 7 Baker IDI Heart and Diabetes Institute, Melbourne, VIC Australia; 8 Department of Medicine, Monash University, Melbourne, VIC Australia; 9 Department of Epidemiology and Preventive Medicine, Monash University, Melbourne, VIC Australia; 10 School of Exercise and Nutrition Sciences, Deakin University, Burwood, VIC Australia; 11 School of Sport Science, Exercise and Health, The University of Western Australia, Perth, WA Australia; 12 Mary MacKillop Institute for Health Research, The Australian Catholic University, Melbourne, VIC Australia; 13 Department of Health Sciences, University of Leicester, Leicester, UK

**Keywords:** Sedentary behaviour, Sit-stand desk, Workplace sitting, Behaviour change, Intervention, COM-B framework

## Abstract

**Background:**

Sitting (sedentary behaviour) is widespread among desk-based office workers and a high level of sedentary behaviour is a risk factor for poor health. Reducing workplace sitting time is therefore an important prevention strategy. Interventions are more likely to be effective if they are theory and evidence-based. The Behaviour Change Wheel (BCW) provides a framework for intervention development. This article describes the development of the Stand More AT Work (*SMArT Work)* intervention, which aims to reduce sitting time among National Health Service (NHS) office-based workers in Leicester, UK.

**Methods:**

We followed the BCW guide and used the Capability, Opportunity and Motivation Behaviour (COM-B) model to conduct focus group discussions with 39 NHS office workers. With these data we used the taxonomy of Behaviour Change Techniques (BCTv1) to identify the most appropriate strategies for facilitating behaviour change in our intervention. To identify the best method for participants to self-monitor their sitting time, a sub-group of participants (*n* = 31) tested a number of electronic self-monitoring devices.

**Results:**

From our BCW steps and the BCT-Taxonomy we identified 10 behaviour change strategies addressing environmental (e.g. provision of height adjustable desks,), organisational (e.g. senior management support, seminar), and individual level (e.g. face-to-face coaching session) barriers. The Darma cushion scored the highest for practicality and acceptability for self-monitoring sitting.

**Conclusion:**

The BCW guide, COM-B model and BCT-Taxonomy can be applied successfully in the context of designing a workplace intervention for reducing sitting time through standing and moving more. The intervention was developed in collaboration with office workers (a participatory approach) to ensure relevance for them and their work situation. The effectiveness of this intervention is currently being evaluated in a randomised controlled trial.

**Trial registration:**

ISRCTN10967042. Registered on 2 February 2015.

## Background

High levels of sedentary behaviour have been consistently linked to increased morbidity and mortality in many epidemiological studies [1–5], although recent research has shown that participating in physical activity may attenuate and even eliminate these links [6, 7]. However, high amounts of physical activity, approximately 60–75 min of moderate-to-vigorous activity per day (more than twice the recommended guidelines in the UK), are likely to be needed [7]. Evidence shows that the majority of the population spend high amounts - around 8–10 h - of their day sitting [8], and self-reported data from the UK shows that 40% of the population are not achieving the current recommended guidelines of 150 min of moderate-to-vigorous physical activity per week [9]. This is likely to be considerably higher when objectively measured with wearable technology rather than by self-report [10]. Therefore, it seems prudent that the amount of time spent sitting should be a concern for the majority of the population. In particular, office workers have been shown to be a highly sedentary population, both inside and outside of work, spending 75% of their workday sitting [11] and approximately 10 h sitting across the whole day on workdays [12]. Furthermore, office workers who are most sedentary at work are also more sedentary outside of work [12].

Reducing occupational sitting time has been the focus of much research in recent years [13, 14]. Interventions have focused on information provision, counselling, policy changes, and making physical changes to the workplace environment, such as providing sit-stand desks. A recent Cochrane review of interventions for reducing sitting time at work found that sit-stand desks led to reductions in sitting of between 30 min to two hours per day [14]. However, studies were only short-term and the quality of the evidence was classified as low to very low quality due to issues such as small sample sizes and non-randomised pre-post designs.

**Fig. 1:**
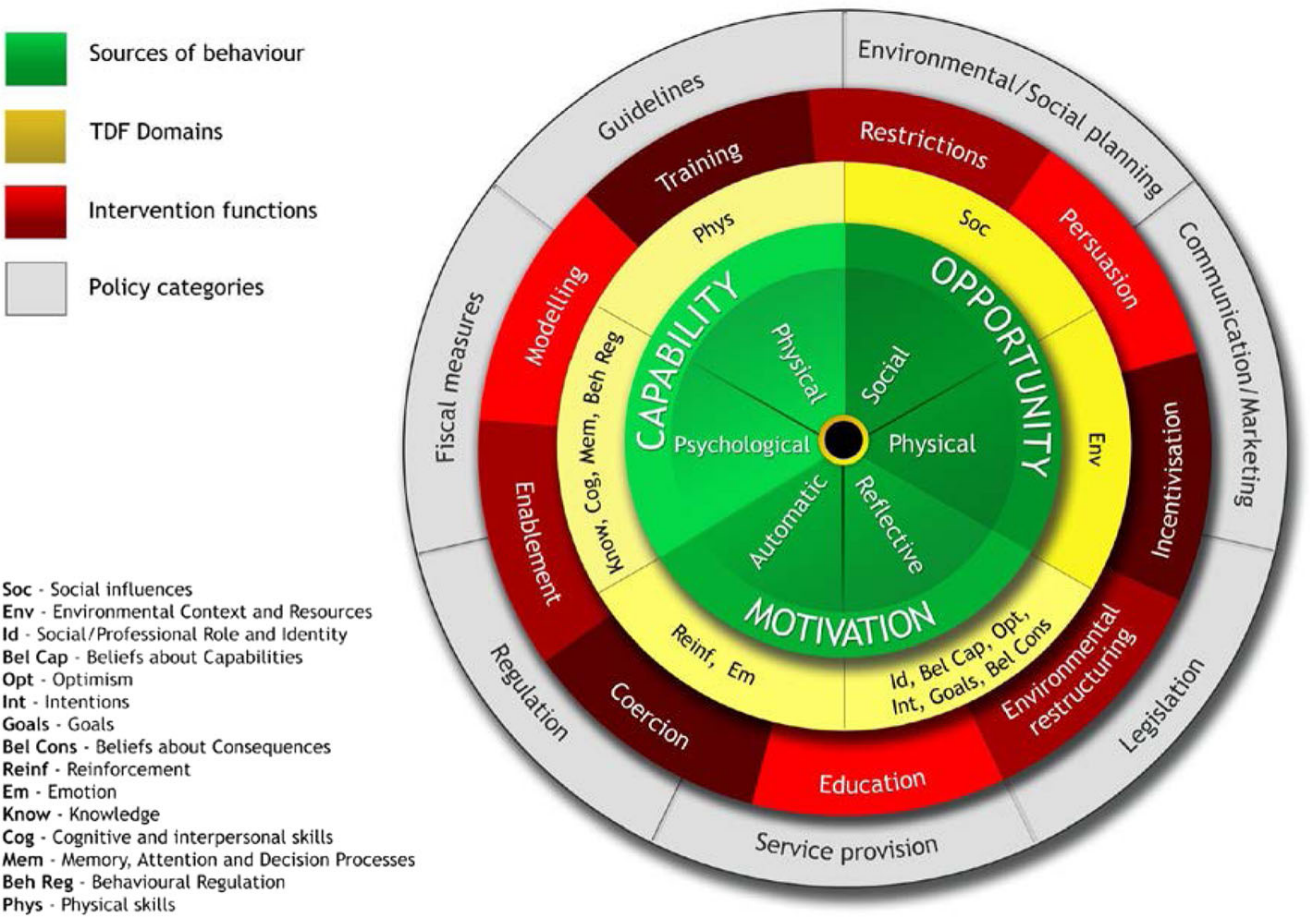
The Behaviour Change Wheel. Green -Sources of behaviour. Yellow -TDF domains. Red – Intervention functions. Grey – Policy Categories. Soc- Social Influences. Env – Environmental Context and Resources. Id – Social/Professional Role and Identity. Bel Cap – Beliefs about Capabilities. Opt – Optimism. Int – intentions. Goals – Goals. Bel Cons – Beliefs about Consequences. Reinf – Reinforcement. Em-Emotion. Cog – Cognitive and interpersonal skills. Mem – Memory, Attention, and Decision Process. Beh Reg - Behavioural Regulation. Phys – Physical skills. Reproduced from: Susan Michie [36]

We designed a workplace intervention (*SMArT Work*: Stand More AT Work) that aimed to address these limitations and which apriori involved height-adjustable workstations to reduce occupational sitting time.. This is being robustly evaluated through a cluster randomised controlled trial [15]. The purpose of the current paper is to describe the systematic process that was employed to develop the intervention components and delivery. As *SMArT Work* aims to change sitting behaviour specifically in the workplace, we used the Behaviour Change Wheel (BCW) [16] and its functions to enhance the development of the intervention (see Fig. 1). The BCW is a comprehensive framework for designing interventions by explicitly integrating behaviour theory to understand and target mechanisms of action within the intervention [17]. The BCW has been developed using expert consensus and a validation process [16]. The wheel has three layers; at its core, it has the COM-B model comprising **C**apability (physical and psychological), **O**pportunity (social and physical) and **M**otivation (automatic and reflective). Michie et al. [16] proposed that people need these three factors to enhance the likelihood of performing the behaviour (B) in question. The COM-B is supported by the Theoretical Domains Framework (TDF) which describes 14 factors from 33 theories of behaviour change that fall under the categories of Capability, Opportunity and Motivation [18]. This allows for a more parsimonious organisation of potentially influencing behaviours than having to deal with multiple, and often complex, theories.

The second layer of the BCW comprises nine intervention functions (Education, Persuasion, Incentivisation, Coercion, Training, Enablement, Modelling, Environmental Restructuring and Restrictions). These are how an intervention might change behaviour, and have been linked to a taxonomy of 93 replicable behaviour change techniques (BCTv1) [19] which are considered ‘active ingredients’ of behaviour change. Each intervention function is likely to consist of several BCTs and any one BCT may serve several functions. The final layer of the wheel comprises seven policy categories that can be used to support the delivery of the intervention functions. Using the structured approach of the BCW, starting with the COM-B model, offers clarity to the process of intervention development and facilitates its subsequent implementation and evaluation [16]. In our protocol article [15], we described the design of the trial to test the effectiveness of the *SMArT Work* intervention. In the present article, we describe the development of the *SMArT Work* intervention using the COM-B model, the BCW functions and the BCT-Taxonomy (v1).

## Methods

**Fig. 2:**
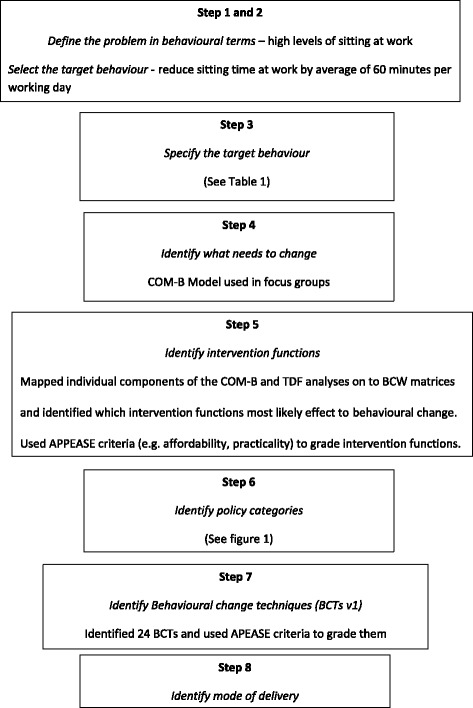
The eight steps of the Behaviour Change Wheel

The study received ethical approval from Loughborough University (Reference Number SSEHS 1751) and approval and authorisation from Research and Development, University Hospitals Leicester NHS Trust (UHL reference 164,119). In the BCW guide, the intervention design method was separated into the eight steps, briefly outlined below, as recommended by Michie et al. [17]. We (BJ, FM, SOC and CE), broadly followed these eight steps in developing *SMArT Work* (see Fig. 2). These were then reviewed by the wider team (SB, TY, DD, LG and MD) and the project steering group (see acknowledgements).

### Step 1: Define the problem in behavioural terms

The first step was to identify the problem behaviour that the intervention addresses (e.g. prolonged/excessive sitting). This included identifying who was performing the behaviours and listing all other behaviours that might influence the problem behaviour.

### Step 2 and Step 3: Select and specify the target behaviour

Outline the new target behaviour (e.g. reduce sitting), who needs to do it, what they need to do differently to achieve change, where and when they need to do it, how often and with whom.

### Step 4: Identify what needs to change

Focus groups or interviews are recommended, using the COM-B model as the basis for discussion, to aid a deeper understanding of behaviours that need to change for the target behaviour to occur. In this study, we conducted focus groups with office-based NHS staff to explore their Capability, Opportunity and Motivation (COM-B) and the Theoretical Domains Framework (TDF) [18] to reduce their sitting time (see below section). With the collected data, the COM-B and TDF] psychological domains that needed targeting in the intervention, for example, knowledge, skills, goals, beliefs about capabilities (see Cane et al.’s paper [18] for the full list and Table 4 for the domains relevant to this study).

### Step 5 and 6: Identify intervention functions and policy categories

The intervention functions (see introduction for a description) to most likely affect behaviour change in the main intervention were selected based on the COM-B and TDF behaviour analyses. The relevant intervention functions were then graded using the APEASE criteria from the BCW guide. These criteria are 1) affordability, 2) practicality, 3) effectiveness and cost-effectiveness, 4) acceptability, 5) side-effects /safety, and 6) equity. How each of these intervention functions could be supported was determined by using the policy categories in the BCW guide (e.g. marketing, guidelines, service provision, etc.).

### Step 7 and 8: Identify behaviour change techniques and mode of delivery

The BCW guide describes how each BCT is linked to the intervention functions. From the list of 93 BCTs, the most appropriate were selected for the intervention that would bring about the desired change (i.e. sitting less at work). In addition, the mode of delivery for each BCT was also selected as part of the implementation plan. Finally, the actual behaviour change intervention activities were identified, designed to be implemented in the *SMArT Work* intervention trial.

### Focus groups

NHS office-based employees from all three hospitals in the locality were invited to take part in COM-B/TDF-based focus groups (step 4 of BCW guide). The research team at the Leicester Diabetes Centre hold a database of office units within the University Hospitals of Leicester NHS Trust. The intervention development study was promoted using the Trust’s intranet system, emails to department managers, and project flyers and posters delivered to appropriate administrative departments. This was followed up with a face-to-face presentation/meeting to discuss the project further. A stratified sample of NHS staff (e.g. employees, managers, gender, job role), were targeted. Interested participants were sent an invitation letter, participant information sheet and a reply slip via email. Those who returned their reply slip to the research team either by email or in person were invited to take part in a focus group and self-monitoring device trial.

Eight focus groups were conducted (ranging in size from 2 to 7 participants) with 39 office-based participants from across three Leicestershire hospitals. All participants worked full-time and 51% reported sitting at work for six hours or more. Over three quarters (79.5%) of the participants were female (see Table 1) and represented financial, procurement, research and clinical-based support services and departments. Focus groups are a form of group interview that use group interaction as part of the method. The researcher facilitates the discussion between participants who share their knowledge and views, exchange ideas and comment on each others’ experiences in ways that are not possible in a one to one interview [20].

**Table 1:** Details of the focus group participants (*n* = 39)

Job type/grade	Sample size (*n*)	Manager/Executive level *N* (%)	Age (range)	Gender (women) *N* (%)	White ethnicity *N* (%)	Education at degree level *N* (%)	Self-reported sitting time hours at work per day (*n* (%))^a^						Device trial *N* (%)
							2–3 h	3–4 h	4–5 h	5–6 h	6–7 h	> 7 h	
Hospital site 1	24	5 (21.0)	20–59	19 (83.3)	18 (75.0)	15 (62.5)	0 (0.0)	1 (4.2)	3 (12.5)	2 (8.3)	5 (20.8)	6 (25.0)	21 (87.5)
Hospital site 2	8	2 (25.0)	30–69	5 (62.5)	7 (87.5)	8 (50.0)	0 (0.0)	0 (0.0)	0 (0.0)	0 (0.0)	5 (62.5)	3 (37.5)	5 (62.5)
Hospital site 3	7	1 (14.3)	30–59	6 (85.7)	7 (100)	7 (100)	1 (14.3)	0 (0.0)	3 (42.9)	2 (28.6)	0 (0.0)	1 (14.3)	5 (71.3)
Total	39	8 (20.5)	20–59	30 (77.0)	32 (82.1)	30 (77.0)	1 (2.6)	1 (2.6)	6 (15.4)	4 (10.3)	10 (25.6)	10 (25.6)	31 (79.5)

^a^All participants reported working 7 h or more per day

The focus group schedule was planned to facilitate discussion on the barriers and facilitators to reducing sitting at work and ascertain which COM-B component(s) and TDF domains should be the primary focus of intervention strategies. Focus groups discussed the following: 1) perceptions of high levels of sitting on health, 2) observations on high levels of sitting in the workplace, 3) perceptions of the barriers to reducing sitting at work, 4) perceptions of facilitators (Capability, Opportunity and Motivation) to reduce high levels of occupational sitting through behaviour change strategies (using the TDF domains as further discussion points), and 5) preferences for height-adjustable workstations. The focus groups were conducted on the site of the participants’ workplace and were facilitated by two trained researchers (SOC and BJ). The discussion schedule was standardised across the different focus groups but with some flexibility to allow for further prompts or discussion between the participants. The researchers refrained from taking part in the discussions but guided the discussions with open-ended questions around the five topic areas outlined above. A summary of the discussions was provided by one of the researchers at the end of the focus groups with the opportunity to clarify or add any missing views. Notes were made at each focus group, along with audio recording. As this was a qualitative study, a formal sample size calculation was not conducted, and focus groups were run until the point of data saturation whereby no new information arose in the last two focus group discussions [21]. Demographic data were collected at the focus groups including age, gender, job type, and working hours via a short questionnaire.

### Readiness-to-change

Prior to the start of each focus group, each participant completed the readiness to change questionnaire. The questionnaire was based on the Community Readiness for Change Handbook [22]. The Community Readiness Model stems from the Transtheoretical Model of Behaviour Change [23], and assesses five dimensions of readiness relevant for reducing desk-based sitting time: *knowledge of efforts, leadership, climate* (prevailing attitudes in the Trust about sedentary work)*, knowledge of the issue* (e.g. health risks linked to prolonged sitting) and *resources* (e.g. funding, staff). Participants responded to each of these dimensions using Likert scales or open responses. Two of the authors (SOC and BJ) scored each dimension from 1 to 9, where 1 = *no awareness* to 9 = *community ownership*. Application of the Community Readiness Model allows you to match any intervention strategies to a population’s level of readiness. A baseline average readiness score was calculated that was used by the team to help further tailor the intervention strategies, whilst a post-intervention score will be measured as part of the RCT evaluation to determine any change in readiness post-intervention.

### Analysis and intervention development

Recorded focus group interviews were transcribed verbatim. Template analysis [24] was used to analyse the focus group textual data. Template Analysis is a method of thematic analysis which uses hierarchical coding in the process of analysing textual data with the flexibility to adapt it to the study [25]. This approach also encourages the researcher to develop themes where the richest data are found in relation to the research question [25]. First, we carried out preliminary coding using *a priori* themes on two focus group transcripts to identify which of the 14 TDF domains played an important role and might facilitate the target behaviour. The emerging themes were then organised into meaningful clusters and an initial coding template was defined. This was then used to ascertain the relevance of each of the COM-B components and the TDF domains to sedentary behaviour, which, in turn, were mapped onto a selection of intervention functions and behaviour change techniques [19]. The template was then applied to a further focus group dataset and refined. The final template was then applied to the full dataset.

### Device testing

Self-monitoring has been identified as one of the most important behaviour change techniques to increase health behaviours [26], including reducing sitting time [27]. With advances in technology, several electronic devices have become available for self-monitoring time spent sitting or lack of movement. It has been suggested that the use of electronic approaches to self-monitoring might lessen the burden of traditional methods (e.g. diaries) and may improve the adherence to self-monitoring and thus indirectly result in greater achievement of behaviour change goals [28].

Thirty-one participants who took part in the focus groups agreed to test the devices identified as possibilities for the study. Four devices were chosen that could monitor and provide feedback on sitting/inactivity: Darma cushion (Darma Inc., CA), Jawbone UP24 (Jawbone, San Francisco, USA), LumoBack (Lumo Bodytech Inc., CA), and Polar Loop (Polar Electro Ltd., UK). Each participant wore between one and three devices at different times and in a random order. Brief written instructions on how to use each device were given. Each participant was asked to set themselves a ‘reduction in time spent sitting’ goal (e.g., 30 min each day) whilst trialling each device. In total, each device was tested by 10 participants for up to a week. For each device, participants completed a questionnaire which asked about the following: battery life, charging, synching data, presentation, navigation and understanding of feedback, ease of use, obtrusiveness and usefulness for monitoring sitting behaviour and encouraging reductions in sitting. Each question was scored on a scale of 1 to 5, with 1 being the least positive and 5 being most positive answer. Focus groups were then conducted with the 31 participants to obtain more detailed feedback and discussion on the usefulness of the devices for self-monitoring sitting behaviour. Template analyses was applied to the textual data from the focus groups using a similar process as described above.

## Results

### Step 1: Define the problem in behavioural terms

Through our own previous empirical work [12, 29] and the existing literature [30], we identified that high levels of workplace sitting was a key behaviour problem and that interventions were needed to reduce sitting time.

### Steps 2 and 3: Select and specify the target behaviour

Our target behaviour was to reduce sitting time at work by an average of 60 min per working day [15], through our main strategy of participants using height-adjustable workstations. Two desk choices were offered to participants in the intervention trial: an electric height-adjustable desk that replaces the participants’ existing desk, or a height adjustable platform, with a two-tier design, which sat on top of the participant’s existing desk (Varidesk.com). Studies of workplace interventions have shown that providing office-based workers with height adjustable desks can substantially reduce sitting time at work [14]. Furthermore, providing an environmental change, such as desks, makes standing and movement more accessible during the working day. This is consistent with the view that health behaviour, and particularly sitting, will be more successfully targeted by making the behaviour easier rather than expecting individuals to become more motivated [31]. Table 2 summarises the target behaviour, who will perform the target behaviour, where they need to do it and with whom.

**Table 2:** Step 3 – Specification of the target behaviour

*What* target behaviour?	Reduce sitting time at work throughout the day for 12 months
*Where* does the behaviour occur?	Work desks of support-staff workers across NHS sites of three Leicester hospitals
*Who* is involved in performing the behaviour?	Desk-bound office workers in any department employed at the sites above

We identified two modifiable target behaviours for intervention development: 1) using prompts or triggers to break-up sitting time, and 2) using strategies to promote regular standing-time. Early development work and existing literature suggests these behaviours are important for reducing sedentary behaviour [30], and that they may be relatively easy to implement and reasonably easy to measure.

### Step 4: Identify what needs to change

All focus group participants completed the readiness to change questionnaire [22] and the results from the six dimensions are presented in Table 3. The overall mean score was ‘vague awareness’. This reflects that participants were aware of what sedentary behaviour meant and that musculoskeletal problems such as back pain may be associated with it. Other health risks associated with sedentary behaviour were not fully understood. The results showed that participants knew that sedentary behaviour at work was an issue; but there was no immediate motivation to do anything about it. These results were explored further in the focus groups where participants discussed how their knowledge of the risks of sedentary behaviour was low. Furthermore, their motivation and ability to reduce sitting were also low. The results from the readiness to change questionnaire were considered when applying intervention functions derived from the BCW process, to ensure that the intervention was delivered at an appropriate level of readiness.

**Table 3:** Community Readiness for Change (*n* = 39)

Dimension	Score*	Level
Knowledge of efforts	3	Vague awareness
Leadership	3	Vague awareness
Climate	5	Preparation
Knowledge of the issue	5	Preparation
Resources	2	Denial/Resistance
Readiness Score	3.6	Vague awareness

^*^Scores range from 1 to 9, where 1 = *no awareness of the risk of sedentary behaviour* to 9 = *community ownership of reducing sedentary behaviour at work*

The themes that emerged from our focus group analyses are presented in Table 4 and illustrated with quotes below. The last theme Capability, Opportunity and Motivation, reflect the three components of the COM-B model - –and findings are presented under each component as sub themes.

**Table 4:** Matrix of links between COM-B model, TDF domains, intervention functions and behaviour change techniques for the SMArT Work intervention

Behavioural analysis using COM-B – barriers and enablers for breaking up prolonged sitting time (step 4)		TDF domains linking to COM-B components (step 4a)	Intervention functions (step 5)	Behaviour Change techniques (BCT v1) (step 7)	Description of intervention strategies
CAPABILITY	*Psychological Capability:* Limited knowledge about the health risks of high levels of sitting (barrier)	*Knowledge* Develop scientific knowledge of the health risks of high levels of sitting and the benefits of reducing sitting time; knowing how to reduce sitting time	Education, Training	*Education:* Information about health consequences; feedback on behaviour; feedback on outcome(s) of behaviour; prompts/cues; self-monitoring of behaviour	*Education, Training, Modelling & Enablement:* An educational seminar at the start of intervention, posters at different stages, goal setting diary with educational messages, demonstration given by researchers on how to use the desk, instruction booklet provided *Environmental Restructuring:* Darma cushion which provides prompts to stand
	Need to notice and remember to stand more	*Memory, Attention & Decision Processes* Know how and when to stand and for how long; and make decisions over tasks that can be conducted whilst standing	Education, Training, Environmental Restructuring, Modelling, Enablement	*Training:* Demonstration of the behaviour; instruction on how to perform a behaviour; self-monitoring of behaviour	
	Limited understanding on how to manage or change own behaviour (goals, self-monitoring) (barrier)	*Behavioural Regulation* Develop skills of goal-setting, action-planning, self-monitoring and breaking prolonged sitting habit	Education, Training, Enablement	*Environmental Restructuring:* Prompts/Cues *Modelling:* Demonstration of the behaviour	
				*Enablement:* Goal setting (behaviour); goal setting (outcome); self-monitoring of behaviour; action planning	
OPPORTUNITY	*Social Opportunity* Perceptions that social norms make it difficult to stand at a desk and work (barrier)	*Social Influences* To provide opportunity to observe colleagues in using a sit stand desk and regularly breaking up their sitting time (i.e. positive role models) by randomising participants by office clusters	Modelling, Enablement	*Modelling:* Demonstration of the behaviour *Enablement:* Social support (unspecified)	*Modelling:* Demonstration by researchers on desk use, clusters using their desks in the same office *Enablement:* Posters updated every three months with motivational quotes from intervention participants
	*Physical Opportunity* To have a height adjustable desk (enabler)	*Environmental Context & Resources* Being able to break up prolonged sitting time at work by having a height-adjustable sit-stand desk	Environmental Restructuring, Enablement	*Environmental Restructuring* and *Enablement:* Restructuring the physical environment; adding objects to the environment	Provide height-adjustable workstations (choice of desktop or full desk)
MOTIVATION	*Automatic Motivation* Staff need simple automatic reinforcement to change habit (enabler)	*Reinforcement* Reinforce routines and habits	Training, Environmental Restructuring; Incentivisation	*Training:* self-reward; habit formation *Environmental Restructuring:* Prompts/ cues *Incentivisation:* Self-monitoring of behaviour; remove aversive stimuli	Sit-stand desk use supported by a paper diary to record daily sitting and standing time, to set short/long-term goals. This is reinforced by the Darma cushion and its App.
	*Reflective Motivation* Beliefs about positive consequences of standing are low (barrier), but beliefs about negative consequences of prolonged sitting is high (enabler).	*Social/Professional Role & Identity* Create individual and group identity that sitting and standing regularly is part of daily work	Persuasion, Modelling,	*Persuasion:* Information about social and environmental consequences; feedback on behaviour; feedback on outcome(s) of behaviour; identification of self as role model; social comparison; *Modelling:* Demonstration of the behaviour	*Persuasion:* seminar, diary, Darma cushion, leaflets/posters, ActivPAL feedback (data from accelerometer provided at individual and group level); coaching sessions
		*Beliefs about Capabilities* Believing that regularly standing at work is achievable will require improved cognitive and self-regulations skills	Incentivisation		
		*Intentions* Develop intentions to sit and stand regularly when working at desk	Education, Enablement	*Incentivisation:* Feedback on behaviour; feedback on outcome(s) of the behaviour	*Incentivisation & Enablement:* Darma cushion feedback via phone app, ActivPAL feedback via researcher; goal setting via diary, coaching sessions *Education:* seminar, posters/leaflets, coaching sessions
		*Goals* Develop goals to break up prolonged sitting by regularly standing when working at desk	Education, Persuasion, Enablement	*Enablement:* goal setting (behaviour); goal setting (outcome); action planning; review behaviour goals; review outcome goals	
		*Beliefs about Consequences* Believing that regularly standing at work is beneficial	Education	*Education:* Information about health consequences	

### Perceptions on high levels of sitting on health

Around two-thirds of the participants recognised sedentary behaviour as a health problem for musculoskeletal issues and obesity. Many participants commented on how their back would hurt from sitting for too long:

*‘When you are sat there for a couple of hours and you look at this, and you, you just slouch, and you start to, your posture goes… and it’s the whole thing’* (participant 2, focus group 9)

However, knowledge about the other risk factors associated with sedentary behaviour was low and only several participants mentioned the risk of diabetes from being too sedentary. Other risks factors were not discussed by participants.

### Observations on high levels of sitting in the workplace

Participants felt there were high levels of sitting in their offices and that sitting was the norm and expectation from the workplace.

*‘They will say you must take breaks every 20 minutes but if you’ve got work, you are not going to get up every 20 minutes and walk around the office for no good reason’* Participant 1, focus group 6)

*‘They just expect you to sit until you basically go home’* (participant 4, focus group 6)

Some participants recognised that whilst it was ‘normal’ to sit for lengthy periods at work, it was not good for musculoskeletal health:

*‘It’s just normal to sit down for such long periods of time. Your body is just not really meant to do that’* (participant 5, focus group 6).

### Perceptions of the barriers to reduce sitting at work

Being absorbed by work was a key barrier as to why participants did not break-up their sitting time. In many cases, wanting to complete work meant that participants were sitting for long stretches of time.

*‘I think quite often you get sort of sucked into what you’re doing, and you know it’s just a case of you want to crack on because you’ve got too much work’* (participant 1, focus group 9.)

Standing at a workstation was currently perceived as unusual as there was no culture for it and participants discussed how if they stood up to work, there would be a reaction from the other workers in their office.

*‘There would be an awful lot of mickey taking…’* (participant 2, focus group 9)

### Capability, Opportunity and Motivation to reduce sitting at work

Participants were encouraged to discuss and suggests ways in which their capability, opportunity and motivation to reduce sitting at work could be supported. Within each component, the 14 TDF domains were raised as discussion points by the researcher as appropriate. The sub-themes related to each component are presented below. Within each sub-theme, the relevant TDF domains are also discussed, presented in parenthesis and in Table 4.

### Capability

Whilst some participants reported that their limbs initially felt stiff when standing and moving after periods of prolonged sitting, they all reported that they could stand and work without any physical problems, but lacked the opportunity to do so. Therefore, *physical capability* of standing and working was not an issue identified from the COM-B model. When considering *psychological capability,* however, participants reported that it was important to understand the health risks of high levels of sitting at work. This suggests that knowledge was an important TDF domain to target in the intervention:

*‘There are a lot of people who are completely ignorant of the fact that when you’re sitting for prolonged periods, there’s bad effects on your health….. so yes, increasing awareness would certainly help’* (participant 4, focus group 4. TDF: Knowledge)

Nearly all participants highlighted that because many people are absorbed by their work and by work deadlines, the ability to remember to stand would be affected (reflecting the Memory, Attention and Decision Processes domain of the TDF). Participants felt prompts or triggers to encourage regular standing would be important to break up sitting time.

*‘Because you’re so engrossed in work, you’re not thinking about what time it is or anything else, so yes, you definitely need something to say its time [to stand]’* (participant 1, focus group 2. TDF: Memory, Attention and Decision Processes)

Being able to self-monitor sitting time would also help participants become aware of their sitting and standing behaviour:

*“Because you don’t realise actually, you think you are having enough breaks but at the end of the day when you see it….*’ (participant 4, focus group 6. TDF: Behavioural Regulation)

### Opportunity

The opportunity of environmental context and resources were discussed, and participants agreed that a height adjustable desk was an important determinant to reduce workplace sitting time. However, despite the literature highlighting the importance of environmental changes to encourage movement (e.g. centrally located printers and bins [29]), this was not relevant for our NHS support workers as waste bins and printers were already centrally organised.

*‘We don’t have a bin each in our room….the nearest recycling point is downstairs on the first floor [so] it has already happened … it’s kind of the way I’m already working’* (participant 1, focus group 3. TDF: Environmental Context and Resources)

Participants were asked how they would find moving around more at work, by for example, speaking to colleagues directly rather than sending an email. Active emails were considered difficult by participants because moving from their desk to speak with colleagues meant that incoming emails would pile up and phone calls would be missed. For some staff roles (e.g. reception) frequently moving away from the desk without staff cover would be impossible.

Social opportunity (TDF: Social Influences) was also identified as an important determinant for whether behaviour change was likely to occur. Being part of a team or culture where everyone implemented the plan together was a key factor in terms of motivating each other to reduce sitting time and in providing social support and practical advice to each other. As this was a cluster randomised controlled trial, the needs for social opportunity were partially met and monitored though our process evaluation.

### Motivation

Participants reported that both automatic (e.g. desires, habits) and reflective (e.g. conscious planning) motivation were important to target in the intervention for behaviour change to occur. In terms of automatic motivation, some participants reported lacking motivation to be more active at work because of their current working habits. Reinforcements (TDF domain) such as having goals to reach and having regular prompts to stand up were seen as important to reduce sitting time and possibly change working habits.

*They talk about taking breaks from your computer but do we do it, no….[having a prompt] would encourage me more as a goal. Well yes, that seems more as a driver for me* (participant 1, focus group 3. TDF: reinforcement)

In terms of reflective motivation, all the participants felt that if everyone in their office implemented plans to break up their sitting time at work they would find it less awkward or embarrassing to stand up as well. This reflected the importance of addressing the Social/Professional Role and Identity and the Social influences TDF domains in the intervention with social opportunity.

*‘And the thing is if it was happening to everybody and everybody was doing that thing it would become normal. So, for people to jump up and down would be normal. Whereas, if it was just one person doing it, they’d say oh’* (participant 4, focus group 4. TDF: Social/Professional Role and Identity; Social Influences).

Intentions to reduce sitting behaviour were important to participants. Making plans to reduce sitting at work considered beneficial and more likely for participants to incorporate standing and movement into their work routine.

*I suppose I could perhaps not fill up my water bottle every day, just go the kitchen and get water as and when’* (participant 2, focus group 5. TDF: Intentions)

Beliefs about Capabilities (TDF domain) was also identified as a key domain to target in the intervention. Whilst all participants understood what the benefits were for standing and moving, very few understood the negative consequences of sedentary behaviour. They agreed that if they had more knowledge about the risks of sedentary behaviour they would be more inclined to want to reduce it.

*‘In our office, one of two of us had maybe, musculoskeletal issues so those people are the ones that I’d say are more aware of [the risks of] sitting …but some people don’t and will have their lunch at their desk. They’d be sitting at their desk all morning and then they will sit at their desk all afternoon’* (participant 4, focus group 7. TDF: Beliefs about Capabilities)

*‘I think if you think about it more, then you’ll think more about your health. There are probably things you could do. But I think until you’ve got the high surface to it then you’re not going to do it’* (participant 2, focus group 5. TDF: Beliefs about Capabilities)

### Device testing

A number of electronic self-monitoring devices were tested by a sub-group of participants (72.4% female). When mean scores were calculated for all questions, the Darma cushion scored the highest (mean 4.1/5), closely followed by the Jawbone (4.0/5), then the Polar Loop (3.7/5) and LumoBack (3.6/5). Scores for each individual question are presented in Table 5. Participants found the Darma cushion to be the most accurate and practical to use, easy to view and understand feedback, and easy to set up. The Darma cushion is placed on the work chair of the participant and an app is used to set the vibration function so that the cushion vibrates after a chosen period of time spent sitting, to prompt the user to stand. The app can also be used to track length of sitting time and to provide information on posture.

**Table 5:** Average mean scores for each question by device (*n* = 31)

Questions	Darma cushion	Jawbone UP24	LumoBack	Polar Loop
Battery life	4.5	4.1	4.6	4.0
Ease of charging	4.6	3.8	4.6	4.1
Syncing data from device to web/app	3.7	3.9	3.4	3.6
Presentation of feedback on web/app	4.1	4.3	3.6	3.8
Navigation of feedback on web/app	4.3	4.2	3.6	3.6
Understanding feedback on web/app	4.1	4.3	3.6	3.6
Overall, how easy would you say the device has been to use?	4.3	4.5	3.7	4.1
How obtrusive has the device been to your daily activities?	3.7	3.8	2.3	3.9
Do you agree that the device has been useful for monitoring your sitting behaviour?	4.2	3.8	3.4	3.3
Do you agree that the device has encouraged you to reduce your sitting behaviour?	3.8	3.8	3	3.4

In the focus group discussions, participants confirmed they liked the features of the Darma cushion and particularly being able to change the intensity and frequency of vibration reminders. Mixed views were reported for the aesthetics of the Polar Loop but, whilst the accuracy was questioned, the feedback on the app was easy to understand. Participants found the Jawbone easy to wear and found the vibration function helpful, but mixed views were reported for the app. Finally, the Lumoback was comfortable, except during hot weather (worn on a belt around the waist directly on the skin), the app was easy to understand but all users reported problems with calibration. Despite the Darma and the Jawbone scoring similarly in the questionnaire, when participants were specifically asked about usefulness for monitoring sitting behaviour and encouraging reductions in sitting, the Darma scored more highly than the Jawbone. Therefore, the most practical and preferred self-monitoring and prompting tool to help reduce sitting was the Darma cushion.

### Steps 5 and 6: Identify intervention functions and policy categories

From the results of the focus group and readiness to change questionnaire, we identified seven intervention functions that were relevant to induce the desired change. The three intervention functions *most* relevant for our intervention were Enablement (increasing means/reducing barriers to increase capability), Education (increasing knowledge or understanding) and Training (imparting skills). The links between the COM-B model, the TDF and the intervention functions are shown in Table 4. Next, out of the seven policy categories listed in the BCW guide as potentially useful for achieving behavioural change, we identified four: 1) Communication/marketing – for example, using printed materials to raise awareness of health risks of sedentary behaviour, 2) Guidelines – such as producing and disseminating sit-stand guidelines to the intervention group; 3 and 4) Environmental/Social Planning and Service Provision – i.e., providing sit-stand desks.

### Steps 7 and 8: Behaviour change techniques and their mode of delivery

We used the APEASE criteria to select the most appropriate BCTs for our seven intervention functions. In total, we identified 24 potentially relevant techniques and translated these into the *SMArT Work* intervention as activities to support behaviour change in our cluster randomised controlled trial (RCT) with NHS administrative and clerical workers (see Table 4). The RCT assessed the effectiveness of the *SMArT Work* intervention over the short (3 months), medium (6 months) and long term (12 months). The primary outcome was to reduce sitting time at work and secondary outcomes were improved work performance, work engagement and well-being (see [15] for further details). The main trial results will be described and published elsewhere. Both implementation and data collection were carried out by the researchers SOC (physical activity specialist) and BJ (background in health psychology and training in using the BCW). The eight most promising BCTs identified by the research team using the above data; and their translated behaviour change activities are described below.

#### Information about health consequences

A face-to-face group seminar was identified as the most effective way to target knowledge about the risks of sedentary behaviour and the benefits of reducing and regularly breaking up sitting. This was delivered at the start of the intervention at the group level. Educational leaflets and wall posters were developed to support the key messages from the seminar and included motivational messages. A separate leaflet was designed that explained how to use the desk/platform, with tips for reducing and breaking up sitting and with a summary of the published guidelines on sitting and standing in the workplace. Both leaflets were aimed at the individual level and were given to the participants at the start of the intervention when the height-adjustable workstations were delivered. The researchers (SOC or BJ) gave a physical demonstration to each participant on how to use their height-adjustable workstation. The information in the posters, aimed at the organisational and group level, were changed at three monthly intervals to sustain interest and motivation.

#### Social support (non-specific) and social comparison and identification as self as role model

The intervention was designed as a cluster randomised controlled trial where offices were randomised into the intervention or control group. For the intervention participants, there were 19 office clusters with an average of 4 participants per cluster. Being part of a group helps to create a social environment of regular standing, and this was reinforced by the researchers at the group seminar and where appropriate, in the one-to-one coaching sessions (see feedback on behaviour), in how an office cluster could for example, reduce their sitting time by taking cues from their co-workers who were standing regularly and/or how they could encourage others in their cluster to reduce their sitting time by self-modelling the behaviour.

#### Goal setting and action planning

To support behaviour change, information about how to set goals for reducing sitting at work and on action planning, was explained in the seminar at the group level. At the individual level, each participant was given a paper-based diary (small A6 ring bound booklet) where they could note their goals, set an action plan of how to achieve these goals and review them regularly.

#### Feedback on *behaviour*

To motivate participants in their progress, the senior researcher (SOC) provided each participant with at least four face-to-face coaching sessions over the course of the intervention and checked progress, reviewed action plans and goals, discussed personal and social/group barriers and how to overcome them, reiterated the benefits of reducing sitting.

#### Feedback on *outcome*

Giving regular feedback to participants on their average sitting, standing and movement times across a short period was important to encourage participants to sit less. The primary outcome for the effectiveness trial was sitting time measured using the activPAL monitor at 12 months. Measures were taken at baseline, 3, 6 and 12 months in the RCT. Shortly after each measurement point, participants were given a summary of their own average sitting (short bouts and prolonged bouts); and standing and stepping time during work hours and for the whole day. Feedback was delivered at the individual level.

#### Prompts/cues and self-monitoring

To support a reduction in sitting and regular breaks in prolonged sitting and habit formation, a Darma cushion was provided to each participant at the start of the intervention when their height-adjustable workstation was delivered. One of the project researchers gave a demonstration of how the cushion and its supporting features worked.

## Discussion

This article describes the systematic development of the *SMArT Work* workplace sitting time reduction intervention. Developing effective workplace interventions to reduce and break up sitting requires identifying the appropriate behaviour change components that can be put in place to support sustainable behaviour change. We used a comprehensive approach for developing theory-based intervention components for *SMArT Work* which apriori involved height-adjustable workstations to reduce occupational sitting time. To develop our *SMArT Work* intervention, we used all the steps outlined in the BCW, used the COM-B model and Theoretical Domains Framework (TDF) to interpret our qualitative results and applied the taxonomy of behaviour change (BCTv1) to select the relevant strategies and design the intervention components. We also used the Community Readiness Model to identify how ready the NHS Trust was for changing their sitting behaviour. The results from this questionnaire showed that the participants had little awareness of the risks of sedentary behaviour and that awareness and education on the health and wellbeing risks of sedentary behaviour was needed.

Using elements of a participatory approach [32] the intervention was informed by office workers working within the organisation where the intervention was due to be delivered and evaluated. Our focus group data found that motivation to change behaviour was low because of current working habits and the work culture of sitting. Participants discussed ways in which sitting at work could be regularly broken up with the use of height adjustable workstations. Suggestions included providing social opportunities where most people in an office would have a height adjustable workstation. These findings are similar to those of De Cocker et al. [33] who also conducted focus groups to explore potential intervention strategies to reduce sedentary behaviour at work. However, our findings are more comprehensive as we explored behaviour change strategies whereby participants considered setting goals and having reminders and regular prompts to stand up as important to break up sitting time. Our findings are also similar to those of Neuhaus et al. [34] but go further in that they recognised the importance of social opportunity and social influence in reducing sitting at work.

The results from our focus groups suggested that from the TDF, knowledge, social identity, intentions, beliefs about capabilities, and self-regulation of behaviour were important to address in our intervention. Our BCT strategies were therefore selected to address these domains and included an educational seminar, leaflets and posters, a goal-setting diary, face-to face coaching sessions and a device that monitored sitting time and provided regular prompts to encourage breaking up prolonged sitting. A review by Gardner et al. [27], on the use of behaviour change strategies to reduce sedentary behaviour found that education, environmental restructuring and self-monitoring were promising strategies. The cushion was selected based on participant feedback on trialling a range of self-monitoring and prompt devices and is a novel element of our intervention. It has been suggested that using this participatory approach of trialling the device may enhance the success of the intervention [32, 35].

To our knowledge, this is one of the first studies to fully utilise the BCW including the APEASE criteria, the COM-B model, the TDF and the BCT to design a sitting reduction intervention. This is a key strength of our study. By using these standardised methods and structured guidance we took a comprehensive and a participatory approach to identify the barriers and facilitators to reducing sitting time at work, and for determining the content and format of our intervention components. The advantage of using an integrative theoretical framework is that it ensures all the necessary elements for our intervention programme are in place to maximise potential benefits. Few workplace sedentary reduction interventions have utilised such a comprehensive approach.

However, there are some limitations. First, we only focused on workers’ behaviour change and not changes in organisational policies and practices which might have been helpful in embedding the intervention across the different hospitals at the end of trial. Second, we may not have identified all of the barriers and potential enablers for reducing sitting time at work as we did not include all key stakeholders in our participatory approach and in our focus groups. Human resources and occupational health may have identified further barriers or enablers to address.

## Conclusions

This study provides an evidence-informed and systematically developed multiple component intervention to reduce sitting at work. The study describes the process of using the BCW, the TDF and the BCT to identify and design behaviour change intervention components to support the apriori height-adjustable workstation. Ten behaviour change intervention components were identified and implemented with the height adjustable workstations in the *SMArT Work* cluster-randomised controlled trial. The process outlined here can be used by researchers to guide a comprehensive intervention development process.

## Abbreviations

APEASE: Affordability, practicality, effectiveness and cost-effectiveness, acceptability, side-effects /safety, equity
BCT: Behaviour change technique
BCW: Behaviour change wheel
COM-B: Capability, opportunity, motivation – behaviour
NHS: National Health Service
SCT: Social cognitive theory
TDF: Theoretical domains framework
